# Interdependence of nutrient metabolism and the circadian clock system: Importance for metabolic health

**DOI:** 10.1016/j.molmet.2015.12.006

**Published:** 2016-01-14

**Authors:** Aleix Ribas-Latre, Kristin Eckel-Mahan

**Affiliations:** The University of Texas Health Science Center at Houston (UT Health), Institute of Molecular Medicine, Center for Metabolic and Degenerative Diseases, 1825 Pressler St., Houston, TX 77030, USA

**Keywords:** Circadian, Metabolism, Nutrients, Synchrony, Nuclear receptors

## Abstract

**Background:**

While additional research is needed, a number of large epidemiological studies show an association between circadian disruption and metabolic disorders. Specifically, obesity, insulin resistance, cardiovascular disease, and other signs of metabolic syndrome all have been linked to circadian disruption in humans. Studies in other species support this association and generally reveal that feeding that is not in phase with the external light/dark cycle, as often occurs with night or rotating shift workers, is disadvantageous in terms of energy balance. As food is a strong driver of circadian rhythms in the periphery, understanding how nutrient metabolism drives clocks across the body is important for dissecting out why circadian misalignment may produce such metabolic effects. A number of circadian clock proteins as well as their accessory proteins (such as nuclear receptors) are highly sensitive to nutrient metabolism. Macronutrients and micronutrients can function as zeitgebers for the clock in a tissue-specific way and can thus impair synchrony between clocks across the body, or potentially restore synchrony in the case of circadian misalignment. Circadian nuclear receptors are particularly sensitive to nutrient metabolism and can alter tissue-specific rhythms in response to changes in the diet. Finally, SNPs in human clock genes appear to be correlated with diet-specific responses and along with chronotype eventually may provide valuable information from a clinical perspective on how to use diet and nutrition to treat metabolic disorders.

**Scope of review:**

This article presents a background of the circadian clock components and their interrelated metabolic and transcriptional feedback loops, followed by a review of some recent studies in humans and rodents that address the effects of nutrient metabolism on the circadian clock and vice versa. We focus on studies in which results suggest that nutrients provide an opportunity to restore or, alternatively, can destroy synchrony between peripheral clocks and the central pacemaker in the brain as well as between peripheral clocks themselves. In addition, we review several studies looking at clock gene SNPs in humans and the metabolic phenotypes or tendencies associated with particular clock gene mutations.

**Major conclusions:**

Targeted use of specific nutrients based on chronotype has the potential for immense clinical utility in the future. Macronutrients and micronutrients have the ability to function as zeitgebers for the clock by activating or modulating specific clock proteins or accessory proteins (such as nuclear receptors). Circadian clock control by nutrients can be tissue-specific. With a better understanding of the mechanisms that support nutrient-induced circadian control in specific tissues, human chronotype and SNP information might eventually be used to tailor nutritional regimens for metabolic disease treatment and thus be an important part of personalized medicine's future.

## 1 Introduction

“You are what you eat” is a phrase often used to describe the compromised metabolic health associated with the excessive intake of food with limited nutrient value. While this association seems obvious, less obvious is that our endogenous circadian clocks may reflect what we eat. In fact, our ability to adjust to jet lag, recover from a sleepless night, or respond to and metabolize medicines prescribed may heavily depend on what we eat and when we eat it. Because evidence to date strongly links our internal clock to metabolism and metabolic health, the effect of nutrient intake on our internal 24-h rhythms has taken a spotlight in the field of metabolism research.

Circadian oscillations are naturally recurring rhythms with a periodicity of approximately twenty-four hours. Most organisms display biological circadian rhythms and in humans, they are fundamental to physiology and behavior. The light–dark cycle is considered one of the most potent zeitgebers (or “time-giver”) driving behavioral preferences and almost all organisms studied to date respond to this circadian cue. Animal studies indicate that other cues, such as food, also drive our internal clocks to a significant extent. Fundamentally, as a consequence of the Earth's rotation on its axis, seasonal and daily environmental changes occur to which organisms must adapt at the metabolic level. Diurnal species, such as humans, carry out their daily activity during the light cycle, while nocturnal species are active during the dark cycle. This activity-rest cycle requires metabolic and physiological adaptation, producing rhythms in processes as disparate as blood pressure, body temperature, cardiovascular efficiency, muscle strength, hormonal secretion in blood, cognitive ability, etc. [1], [2], [3], [4]. While anticipation of the changing environment is controlled to a large extent at the level of the brain, where light activates the central clock (the suprachiasmatic nucleus, or SCN), peripheral clocks also host circadian rhythms [5], but respond predominantly to cues other than light. More specifically, nutrient input is a critical and primary driver of several peripheral clocks, such as the circadian clock in the liver [6], [7], [8], [9] and, pending its composition and the timing of administration, can even usurp the local clock, preventing synchronization with the central pacemaker and potentially disrupting synchronization with other peripheral clocks. Becoming more apparent is that nutrient sensing by the clock in different tissues is a powerful mechanism by which tissues maintain or acquire the energy balance necessary to carry out their physiological roles. A large part of this nutrient sensing involves the timing of nutrient input, a topic which has been comprehensively reviewed in several recent reviews [1], [10], [11]. Thus, the main focus of this review will weigh heavily on some of the most recent studies looking at sensing by the clock of specific nutrients or groups of nutrients as well as some of the epidemiological studies highlighting links between the human circadian clock and nutrient metabolism.

## 2 Molecular basis for circadian and metabolic interactions

Circadian rhythms are supported at the cellular level by a wide range of complex molecular pathways and specific oscillatory enzymes. Nonetheless, from a basic point of view, a circadian clock system is shared among species worldwide [12]. The use of omic technologies has made it possible to ascertain the circadian patterns of a significant number of transcripts, proteins and metabolites that drive cellular rhythmicity. High-throughput transcriptional studies using mouse tissues have revealed that at any given point in time in a single tissue, up to a tenth of all mammalian genes exhibit 24-h variations in mRNA levels (reviewed in Ref. [13]). However, recent studies demonstrate that a much larger percentage of genes oscillate in at least one tissue throughout the body [14], promoting the idea that most genes can oscillate in expression depending on the environment [15]. These transcripts include genes controlling processes as widespread as mitochondrial oxidative phosphorylation, carbohydrate metabolism and transport, lipid biosynthesis, adipocyte differentiation, and cholesterol synthesis and degradation [14], [16], [17], [18], [19], [20], [21]. Similarly, additional studies looking at protein regulation throughout the circadian cycle reveal that approximately 20% of the proteome in liver and SCN [22], [23] is subject to circadian control with some posttranslational modifications also cycling in a circadian manner [24], [25]. A significant fraction of the oscillating proteins in a cell is devoid of oscillations at the mRNA level [25]. Thus cellular circadian oscillations take place at several levels of cell function and at several stages in the process of a gene being expressed. Like oscillating gene transcripts, many of the oscillatory proteins within the cell comprise members of various metabolic processes such as urea formation, sugar metabolism and mitochondrial oxidative phosphorylation [22], [23]. Metabolite profiling studies have added additional complexity to the picture of circadian clock-controlled metabolic function. Studies in murine animals show that many metabolites involved in amino acid, carbohydrate, lipid, nucleotide and xenobiotic metabolic pathways, oscillate in liver [26], muscle [27] and plasma [28], whereas 15%–70% of the metabolome in humans exhibits circadian variation depending on whether rhythmicity in energy intake and the sleep/wake cycle is maintained [29], [30]. Overlapping data from various omic studies demonstrate that circadian rhythms are extremely zeitgeber-responsive and specific. For example, when comparing metabolite or transcript oscillations in the liver of mice with different genetic backgrounds or on different diets, it is revealed that many oscillating events are not shared [15]. Furthermore, comparing oscillations across tissues of the same species reveals that many oscillations are tissue specific [14], [15], [17], [31]. Many of the core clock genes oscillate across tissues or species, but many metabolic oscillations are highly dependent on the environment. Thus, the current understanding of cellular circadian rhythms throughout an organism is that while the core clock genes are oscillating in most tissues and in the midst of enormous environmental pressures, metabolic circadian oscillations are strongly shaped by the environment [14], [15].

**Figure 1:**
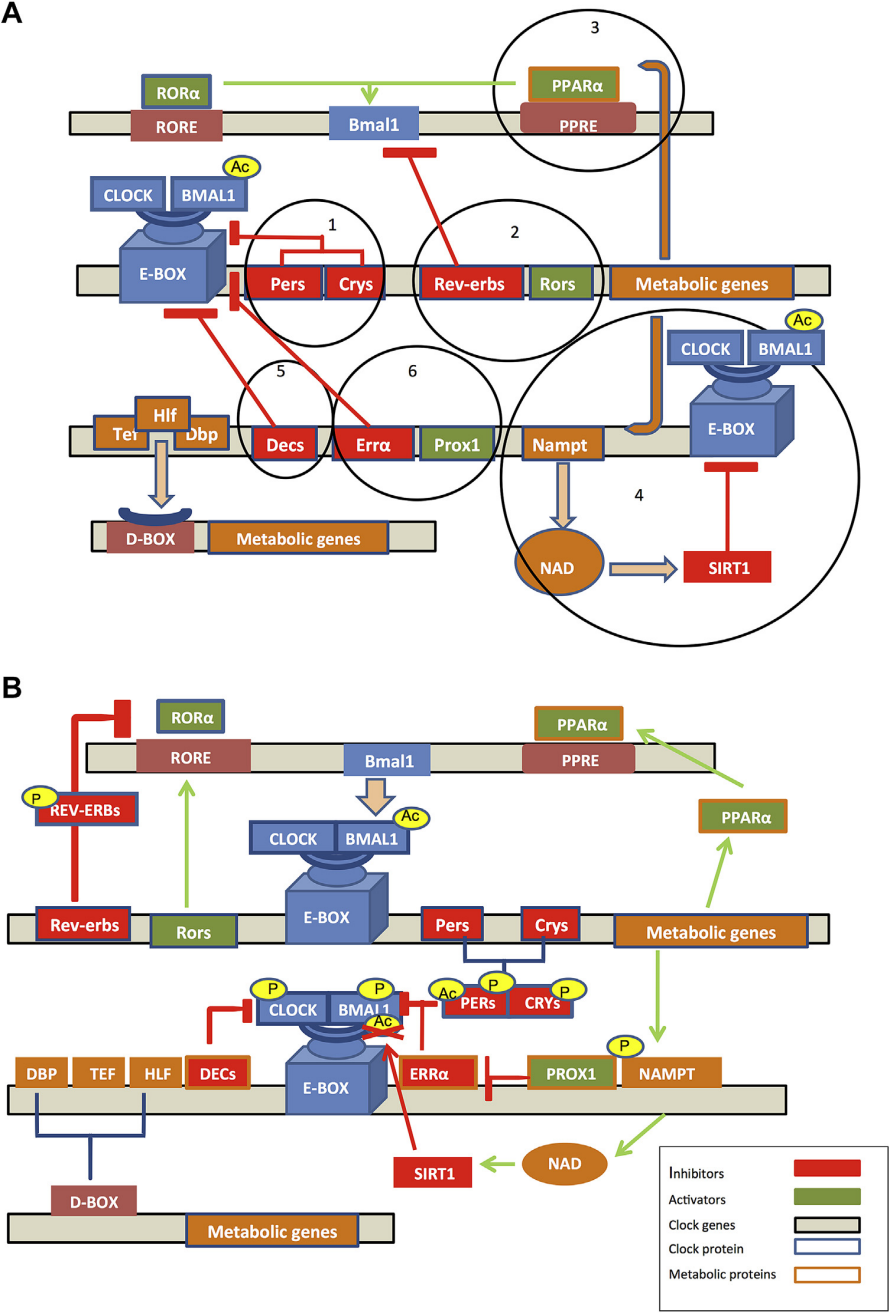
**The molecular clock at the transcription and post-translational level**. (A) The molecular circadian clock is composed of six interrelated transcription–translation feedback loops, with the CLOCK-BMAL1 heterodimer providing the central transactivation at E-box-containing target genes. Loop 1: PER and CRY proteins dimerize and inhibit the activity of CLOCK:BMAL1 heterodimer in the nucleus. Loop 2: The nuclear receptors ROR and REV-ERB both compete for a binding site within the response element (RORE) of the *Bmal1* promoter and activate or repress its transcription, respectively. Loop 3: PPARα activates the transcription of *Bmal1* by binding to the PPARα response element (PPRE) located in the *Bmal1* promoter. Loop 4: NAMPT provides negative feedback by modulating SIRT1 activity via an increase in NAD^+^ levels. Loop 5: DEC1 and DEC2 transcription factors inhibit the CLOCK:BMAL1 activity by direct binding. Loop 6: The nuclear receptor ERRα specifically down-regulates *Bmal1* expression, while its co-repressor PROX1 alleviates its repression. (B) Oscillatory post-translational events of key circadian proteins have important regulatory roles in the TTFL [105]. BMAL1 [106] is acetylated by CLOCK and both BMAL1 and PER2 are subjected to deacetylation by SIRT1. In the case of BMAL1, deacetylation leads to repression of target gene expression [106] while PER2 deacetylation by SIRT1 leads to its degradation [56]. Phosphorylation of BMAL1 by PRKCA results in inhibition of CLOCK:BMAL1 transcriptional activity [108], while phosphorylation of BMAL1 by CK1ɛ and GSK3β also regulates BMAL1 activity [109], [110]. CK1ɛ-mediated phosphorylation activates BMAL1 while GSK3β-mediated phosphorylation prepares it for further degradation. GSK3β also phosphorylates and stabilizes CRY2 [111], PER2 [112], REV-ERBα [113] and CLOCK [114]. PERs and CRYs families are phosphorylated prior to ubiquitination and degradation [43], [44], [45] while NAMPT autophosphorylation increases its enzymatic activity.

The core circadian clock system in mammals depends on a central clock located in the hypothalamic suprachiasmatic nucleus (SCN), and on “peripheral” clocks spread throughout the anatomy [32], [33]. Rhythmicity at the level of the SCN is extremely complex [34], [35] and has two essential functions systemically: integrating direct photic input from the retina through the optic nerve and maintaining the communication among the different clocks through endocrine signals and nerve impulses [36]. As the SCN provides both integration and primary coordination of peripheral clocks throughout the body, it is known as the “master clock”, or “pacemaker” in mammals [37]. In most organisms in which the molecular clock mechanism has been investigated, a common model has been observed across cells, be it those of the central pacemaker or those of the periphery: a transcription–translation feedback loop (TTFL) [38]. In mammals, the positive limb of the TTFL is comprised of the transcriptional activators, the circadian locomotor output cycles kaput (CLOCK) and brain and muscle ARNT like protein 1 (BMAL1). These clock core genes encode bHLH-PAS (basic helix–loop–helix; Per-Arnt-Single) proteins that after their own heterodimerization initiate transcription by binding to specific DNA elements like E-boxes (5′-CACGTG-3′) and E’-boxes (5′-CACGTT-3′) in the promoters of target genes. Loss of either BMAL1 or CLOCK and NPAS2 (a paralog of CLOCK), eliminates functionality of the TTFL altogether and thus circadian rhythms in animal physiology and behavior [39], [40], [41]. CLOCK:BMAL1 target genes can be metabolic genes which do not directly feed back onto the TTFL or they can be so-called “clock genes”, which feed back directly into the clock's TTFL as CLOCK:BMAL1 activity inhibitors or activators [38]. The CLOCK:BMAL1 target genes include the Period (*Per*) and Cryptochrome (*Cry*) genes, which ultimately reach critical protein concentrations, dimerize, and inhibit the subsequent activity of the CLOCK:BMAL1 heterodimer in the nucleus [42]. Degradation of the negative limb proteins PER and CRY is required to initiate of a new cycle of transcription. Casein kinase (CK)1ɛ and CK1δ phosphorylate the PER proteins, which is necessary for their ubiquitination and degradation by β-transducing-repeat-containing protein (βTrCP) and 26S proteasome respectively [43]. CRY1 is phosphorylated by 5′ AMP-activated protein kinase 1 (AMPK1) [44] and CRY2 by a sequential dual-specificity tyrosine-(Y)-phosphorylation regulated kinase 1A(DYRK1A)/glycogen synthase kinase 3beta (GSK-3β) cascade [45], which targets it for ubiquitination and degradation by F-Box And Leucine-Rich Repeat Protein 3(FBXL3) [46], [47], [48], [49]. In addition, the active CLOCK:BMAL1 heterodimer promotes the transcription of the nuclear receptors retinoic acid-related orphan receptor alpha (*Rorα*) and the nuclear receptor subfamily 1, group D (*Nr1d1*), also known as reverse erythroblastosis virus alpha (*Rev-erbα*), its own activator and repressor, respectively. These important nuclear receptors both compete for a binding site within the response element (RORE) into the *Bmal1* promoter, generating another loop of regulation [42]. Overall, the molecular circadian clock is composed of six interrelated transcription–translation feedback loops (Figure 1A) that oscillate around the circadian cycle depending on external demands or modulators.

## 3 Clock-controlled metabolic genes

The number of clock-controlled genes (i.e. genes transcriptionally controlled by CLOCK:BMAL1 via E-box regulation) is extensive. Therefore, we propose a classification of these genes according to their bidirectional clock regulatory functions at the level of interaction with CLOCK and BMAL1 at their cognate E-box target sites (Table 1). Listed genes are all validated CLOCK:BMAL1 target genes, bound directly by the heterodimer [25]. (Here we classify gene targets according to whether, once expressed, they feed back to affect the function of one of the TTFL circadian loops.) The majority of metabolic CLOCK:BMAL1 target genes do not exert a direct regulatory role on the molecular clock. These targets include metabolic genes such as aminolevulinic acid synthase 1 (*Alas1*), plasminogen activator inhibitor-1 (*Pai-1*) or thyroid hormone receptor alpha (*Trα*), which play important output roles in heme biosynthesis and vascular or cardiovascular function, respectively (reviewed in Refs. [50], [51]). Other clock-controlled genes that are direct CLOCK:BMAL1 targets but that do not exert a direct regulatory role on the TTFL include thyrotroph embryonic factor (*Tef*) and hepatic leukemia factor (*Hlf*), which have important regulatory functions activating downstream metabolic target genes through direct binding to D-boxes [52]. Interestingly, *Dpb* binds to D-elements in the promoter of *Per1* [53], and thus feeds back into the TTFL by controlling the negative arm.

Alternatively, there is a group of metabolic CLOCK:BMAL1 target genes that possess direct regulatory feedback properties via one or more of the loops described in Figure 1. Examples of such targets include peroxisome proliferator-activated receptor alpha (*Pparα*), nicotinamide phosphoribosyltransferase (*Nampt*), *Dec1*, *Dec2*, estrogen-related receptor alpha (*Errα*) and proper homeobox 1 (*Prox1*).

In addition to RORα, PPARα is also a positive regulator of *Bmal1* expression and thus functions as a bidirectional clock regulatory protein by binding to a PPARα response element (PPRE) located in the *Bmal1* promoter. BMAL1, in turn, is an upstream regulator of *Pparα* gene expression, producing an additional regulatory feedback loop for peripheral clocks (Figure 1A, loop 3) [54].

Another gene with bidirectional control is the CLOCK:BMAL1 target *Nampt* [55], [56], which is also the rate-limiting enzyme that converts Nicotinamide (NAM) to Nicotinamide Mononucleotide (NMN), a key reaction required for the intracellular salvage of Nicotinamide Adenine Dinucleotide (NAD^+^) [57]. NAD^+^ is a key molecule in metabolism, activating a number of enzymes and transcriptional factors involved in multiple metabolic pathways. The NAD^+^-dependent sirtuin deacetylase, SIRT1, is one such factor, which binds directly to CLOCK:BMAL1 and affects its transactivating activity. Thus, BMAL1 activation of *Nampt* generates an additional negative feedback loop (Figure 1A, loop 4), coupling cellular metabolites and their targets directly to the core clock TTFL [58], [59].

*Dec1* and *Dec2* are also metabolic CLOCK:BMAL1 target genes implicated in cellular differentiation among other processes. Similar to PER and CRY-mediated inhibition of the CLOCK:BMAL1 heterodimer, DEC1 and DEC2, both basic helix–loop–helix transcription factors, bind directly to CLOCK:BMAL1, inhibiting its activity (see Figure 1A, loop 5) [60]. The promoters of *Dec1* and *Dec2* contain both E-box and RORE elements, providing an additional regulatory check point for the TTFL [61].

Other inhibition of CLOCK:BMAL1 activity comes from the nuclear receptor ERRα. ERRα specifically down-regulates *Bmal1* expression (Figure 1A, loop 6), and PROX1 blocks this repression. The interplay between ERRα and PROX1 affects the circadian robustness of some clock target genes including *Per2*, *Cry1*, *Rev-erb-α* and *Rev-erb-β* is important considering that ERRα has been shown to connect energy metabolism to the clock machinery in part via its additional transcriptional control over metabolic gene networks [62].

Although *Bmal1*, *Clock*, *Per1*, *Per2*, *Per3*, *Cry1*, *Cry2*, *Rorα*, *Rorβ*, *Rorγ*, *Rev-erb-α* and *Rev-erb-β* comprise the specific group of clock genes essential for TTFL oscillations, they also possess specific functions in regulating metabolic homeostasis according to studies carried out on a variety of global and tissue-specific knockout mice [39], [41], [63], [64], [65], [66], [67], [68], [69], [70], [71], [72], [73], [74], [75], [76], [77], [78], [79], [80], [81], [82], [83], [84], [85], [86], [87], [88], [89], [90], [91], [92], [93], [94], [95], [96], [97]. Studies have revealed both direct [98] and indirect [68] actions of the clock genes in metabolic pathways. PER2 for instance, can interact or compete with other nuclear receptors, thus regulating rhythmicity in target gene expression [62], [68]. REV-ERB-α directly regulates gluconeogenic enzymes like glucose-6-phosphatase (*G6Pase*), phosphoenolpyruvate carboxykinase (*Pepck*), the nuclear receptor heme binding protein (*Shp*) and nuclear factor interleukin 3 (*Nfil3*) (also known as *E4bp4*) through RORE [99]. In addition, rate-limiting steps of fatty acid oxidation, fatty acid synthesis and cholesterol and bile acid biosynthesis are also under circadian control in the liver, as reflected by cycling in the mRNA or protein levels of the fatty acid transporter carnitine-palmitoyl transferase 1 (CPT-1), the membrane-bound transcription factor sterol regulatory element-binding protein (SREBP)-1c and the rate-limiting enzymes 3-hydroxy-3-methyl-glutaryl-Coenzyme A reductase (HMGCR) and cholesterol 7α-hydroxylase (CYP7A1) [100], [101]. Such cycling poises the body to synthesize lipids, emulsify fats, or transport and oxidize lipids at the right time relative to the eating cycle. These examples may explain in part the close relationship between the circadian clock system and metabolism and serve as a context for the epidemiological studies showing links between the circadian clock in humans and energy balance. It is likely because many of these metabolic oscillations are driven by the eating schedule, uncoupling energy intake rhythms from the environment promotes obesity in rats and mice [102], [103]. This data correlates with human studies showing association between rest phase energy intake and obesity [104].

Approximately half of the rhythmic proteins identified in the mouse liver cannot be explained by the rhythmicity of mRNAs, suggesting that translation and/or protein stability might play a pivotal role in controlling rhythmic protein accumulation [25]. Indeed, oscillatory post-translational events of key circadian proteins have been previously identified to have important regulatory roles [105]. Furthermore, it is a point of intersection between metabolism and the clock as such modifications often rely on oscillations in metabolite substrates that are derived from intermediary metabolism. Related to their activity, BMAL1 [106] is acetylated by CLOCK while PER2 and BMAL1 are both subjected to deacetylation by SIRT1. In the case of BMAL1, deacetylation leads to repression of target gene expression [106] and, in the case of PER2, deacetylation by SIRT1 leads to its degradation [56]. As SIRT1 directly inhibits the CLOCK:BMAL1 activity, SIRT1-induced PER2 degradation may provide a necessary compensatory effect on CLOCK:BMAL1 target gene expression [107].

In addition to acetylation, BMAL1 can also be phosphorylated by protein kinase C alpha (PRKCA), following stimulation by Receptor of activated protein kinase C1 (RACK1). The BMAL1-RACK1-PRKCA complex is rhythmically assembled during the negative phase of the molecular circadian cycle, resulting in an inhibition of CLOCK:BMAL1 transcriptional activity [108]. Moreover, BMAL1 can also be phosphorylated by CK1ɛ and GSK3β [109], [110]. GSK3β targets several circadian proteins including CRY2 (which leads to its stabilization) [111], PER2 [112], REV-ERB-α [113] and CLOCK [114] (Figure 1B). Because GSK3β, a key molecule in the insulin signaling pathway, targets several of the clock proteins, it appears to be a key player in clock control. Indeed, small molecule inhibitors of the protein have been found to induce period shortening of the clock in cells [115]. While additional posttranslational modifications at clock and clock accessory proteins take place and can be activating or deactivating depending on the context, suffice it to say that this level of circadian regulation is essential for rhythmicity in a number of cellular events [43], [44], [45], [116], [117]. For example, protein synthesis is directly affected by rhythmic BMAL1 phosphorylation. BMAL1 is a substrate of ribosomal S6 protein kinase 1 (S6K), and its phosphorylation by S6K specifically allows it to interact with translational machinery in a circadian manner and thus promote cyclic protein synthesis [118]. O-linked β-D-N-acetylglucosamine (O-GlcNAc) modification of CLOCK, BMAL1, and PER2 also takes place in the cell [119], [120] and couples hexosamine biosynthesis to rhythmicity in gene expression by altering the stability of these TTFL proteins.

Interestingly, some part of rhythmically expressed metabolic genes appear to oscillate independently of the TTFL, and have no apparent CLOCK:BMAL1 binding site, suggesting the importance of indirect transcriptional and posttranscriptional regulation [9], [52], [121]. Sometimes, these types of oscillations can be driven by zeitgebers which induce rhythmicity in nutrient-responsive factors such as PPARγ and SREBP-1C [9], [92], [121], [122]. These oscillations provide an additional method of nutrient sensing that does not directly rely on proteins of the TTFL.

## 4 How does the clock respond to a change in energy state?

Sometimes, tissues use tissue-specific mechanisms to carry out metabolic oscillations in response to environmental demands [95]. But more often than not, tissues use common signaling molecules in a temporally unique way to carry out their metabolic functions. Table 2 lists many of the primary functions of several metabolic tissues in addition to listing genes associated with the pathway that either oscillate *in vivo* or are direct targets of one or more of the TTFL proteins *in vivo* [14], [25]. Examples of proteins that may provide such responses across multiple tissues are the sirtuin family of deacetylases, including SIRT1, SIRT3, and SIRT6 as well as the protein kinase AMPK.

SIRT1 is a key regulator of several metabolic processes including gluconeogenesis, lipid metabolism, and insulin sensitivity [142] and targets several factors involved in the maintenance of nutrient flux, including Peroxisome Proliferator-Activated Receptor gamma (PPARγ), Peroxisome Proliferator-Activated Receptor Gamma Coactivator 1-alpha (PGC-1α), Forkhead Box Protein O1 (FOXO1), Transducer of Regulated CREB Protein 2 (TORC2), Signal Transducer and Activator of Transcription 3 (STAT3) and SREBP-1c, among others [143], [144]. Like SIRT1, SIRT6 is sensitive to cellular NAD^+^ levels and can bind to the CLOCK:BMAL1 heterodimer directly to modulate target gene expression [121]; but unlike SIRT1, SIRT6 appears to exert its chromatin effects in part via its sensitivity to specific fatty acids (FFAs) like myristic, oleic, or linoleic acid [145]. Located in the mitochondria, SIRT3 is important in fatty acid oxidation during fasting and provides rhythmic control in part via its ability to deacetylate mitochondrial proteins directly [146]. Thus, the NAD^+^-sensitive sirtuins proteins provide an example of a somewhat ubiquitous method by which cells can fine-tune their clock based on the energy state of the cell.

Another key mechanism by which nutrients and energy status help the clock keep time is via AMPK. AMPK detects changes in cellular AMP:ATP ratios during the rest and activity cycle. As a detector of this ratio, it promotes the rhythmic activation of metabolic pathways in response to increasing AMP levels. The molecular targets of AMPK cover a wide range of metabolic processes like glucose uptake, glycogen and protein synthesis, glycolysis, gluconeogenesis and fatty acid synthesis or oxidation [147].

**Figure 2:**
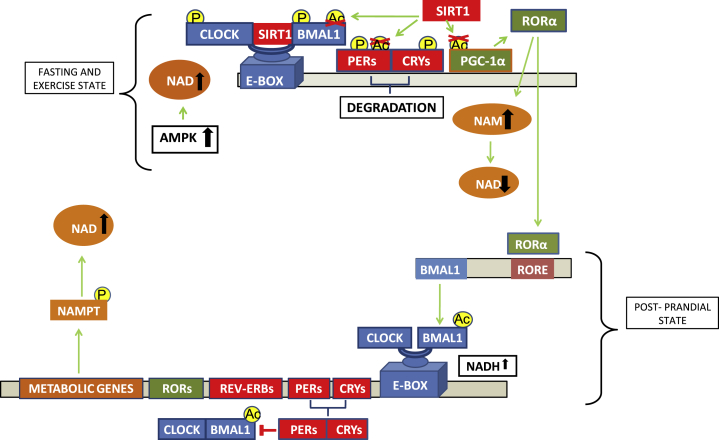
**The molecular clock is sensitive to the energy state**. When cellular energy is low, as under fasting or exercise situations, AMPK is activated by an increase in the AMP/ATP ratio. AMPK induces CRY and CKIɛ phosphorylation (thereby controlling PER degradation) and indirectly activates SIRT1 via increases in NAD^+^ levels [149]. SIRT1 activation results in BMAL1 and PER protein deacetylation. Additionally, SIRTs deacetylates and activates PGC-1α, which co-activates RORα. As a consequence of NAD+-dependent sirtuin activity, nicotinamide (NAM) levels increase, NAD^+^ levels decrease, and SIRT1 activity is downregulated [151]. Increased levels of NADH, such as occurs postprandially, induce CLOCK:BMAL1 binding and activation of target genes as well as BMAL1 acetylation. Over time, PERs and CRY proteins dimerize and bind to CLOCK:BMAL1 and PER is acetylated [151].

NAD^+^ and AMP influence the clock in very direct ways. For example, the DNA-binding activity of the CLOCK:BMAL1 and NPAS2:BMAL1 heterodimers themselves are regulated by the redox state of NAD^+^ cofactors. The reduced forms NAD(H) and NADP(H) strongly enhance DNA binding, while the oxidized forms NAD^+^ and NADP^+^, inhibit it [148]. Thus, there exists substantial crosstalk and feedback within the clock at the level of the energy-related metabolites NAD^+^, NAD(H), and AMP so that when the external energy contribution is low as under fasting or exercise situations, AMPK is activated. AMPK induces CRY and CKIɛ phosphorylation (thus also indirectly controlling PER degradation) and indirectly activates SIRT1 via increases in NAD^+^ levels [149]. SIRT1 activation results in BMAL1 and PER protein deacetylation (i.e. BMAL1 inactivation and CRY:PER destabilization and further degradation). Additionally, under this situation, SIRT1 also deacetylates and activates PGC-1α [150], which co-activates RORα. As a consequence of NAD^+^-dependent sirtuin activity, NAM levels increase progressively, inhibiting in turn NAD^+^ formation and therefore, SIRT1 activity [151]. On the other hand, increased levels of NADH, as observed in a postprandial state induce CLOCK:BMAL1 E-box binding, BMAL1 acetylation and target gene transcription. Over time, PER:CRY binds to CLOCK:BMAL1 and PER is acetylated [151] (Figure 2). Therefore, the NAD(P)^+^/NAD(P)H balance depends on the metabolic cell state and the metabolic cell state depends on the balance between energy intake and energy demands. In summary, the entrainment of the TTFL in a particular cell depends directly on the redox state of the cell [151].

## 5 Feeding and the clock

Many studies have reported the impact of the fasting–feeding cycle on the circadian system through different designs of limited food availability in a period of 24 h (reviewed in Refs. [10], [152]). While data in this area is still somewhat limited, one study showed that fasting during the early activity phase in rodents may increase body weight and lipogenesis concomitant with an increase in gene expression amplitude of pro-lipogenic transcription factors like *Srebp-1c* and *Ppara* in mouse liver [153]. Some human studies support this trend. For example, one study looking at young men who routinely skip breakfast showed that breakfast skippers had an increase in both triglyceride and LDL cholesterol levels in the serum compared to subjects who ate the first of their three meals in the morning [154]. With regards to the clock machinery itself, studies in rodents show that initiation of food intake during the wake phase following a prolonged fast, causes a rapid induction of *Per2* and *Dec1* expression in the rodent liver. One hour following food intake initiation, the *Per1*, *Per2* and *Dec1* mRNA levels increase, while *Rev-erb-α* mRNA levels decrease [155]. Postprandial increases in insulin levels after the breakfast could in part explain this modulation, as injection of insulin induces a rapid change in the *Per1*, *Per2*, *Dec1* and *Rev-erb-α* relative expression not only in liver but other insulin-sensitive tissues like the muscle and the white adipose tissue, an effect which is blocked after the addition of specific inhibitors for MAPK and PI3K [156]. Restricted feeding (RF), where calories are not limiting but confined to a discrete temporal window, can also affect the clock at the level of the TTFL. RF entrains circadian oscillations in peripheral tissues, (reviewed in Refs. [1], [10]). Remarkably, under these conditions, the degree of phase shift varies among different organs, without affecting the clock rhythms in the SCN, thereby uncoupling the phase of peripheral clocks from that of the central clock [6]. (Of note, RF is not similar in this regard to caloric restriction, which can affect the phase of circadian rhythms in the central pacemaker [157]). Additional information regarding the profound effects that restricted feeding can have on the clock and metabolism is reviewed in Ref. [10].

**Figure 3:**
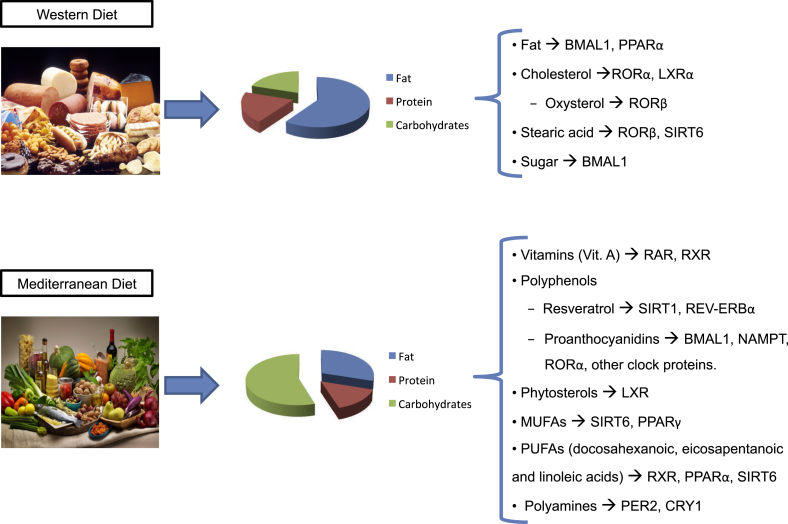
**Diet composition produces diverse zeitgebers for the clock**. Typical food items for the so-called “western diet”, which is generally composed of foods high in saturated fat combined with high sugar content, and a so-called “Mediterranean diet”, which is generally considered to contain a higher percentage of plant-based foods and a substitution of some saturated fats with mono and poly-unsaturated fats. Each of these diets produces macromolecules and metabolites known to function as zeitgebers for the circadian clock in various tissues and cell types. While lipids and cholesterol are known to modulate PPARγ, RORα, LXR, and RORβ, fats (particularly those which affect insulin sensitivity over time), and high glucose likely modulates BMAL1 activity in a GSK3β-dependent manner. Stearic acid has been observed to modulate the sirtuin protein SIRT6 (which binds directly to CLOCK:BMAL1). Under different dietary conditions, there is an increase in potential clock zeitgebers including vitamin A (known to activate RAR and RXR), polyphenols such as resveratrol (activator of SRIT1) and proanthocyanidins (regulators or modulators of *Bmal1*, *Nampt*, and several other clock genes). Additional ligands or modulators of clock-related proteins or rhythmic nuclear receptors include polyamines (regulators of the PER2:CRY1 complex), MUFA and PUFAs (modulators of SIRT6, PPARγ, RXR, and PPARα), and phytosterols (modulators of LXR).

A variety of studies have begun to address how specific diet components can modulate the circadian clock. Diets vary substantially in the type and amount of macro and micronutrients that can be used as zeitgebers for the clock (Figure 3). The case of fat as a component has been studied through rodent models of diet induced obesity. Interestingly, in some cases, a high-fat diet (HFD) administered *ad libitum* to animals results in increased daytime activity and a concomitant increase in caloric consumption during the 12-h light phase compared to control chow-fed animals [158], [159], [160], [161]. Interestingly, HFD can lengthen the circadian period as measured by locomotion analysis and induce a phase advance in the liver clock [161], [162], [163]. In the cases where HFD induces an increase in rest phase energy intake, rhythmic energy intake can be quickly restored by returning the animals to low fat chow [162]. Interestingly, *Clock* mutant mice are resistant to the HFD-induced period lengthening that is sometimes observed in wild-type control conditions [163].

The human clock also appears to be sensitive to dietary manipulations at the level of macronutrient content. In a study designed to test the impact of a low carbohydrate/high fat diet vs. a low fat/high carbohydrate diet on the human clock, it was found that a switch from a low carbohydrate/high fat diet to a high fat/low carbohydrate diet produced a phase delay in salivary cortisol levels (indicative of a longer period centrally), and altered gene expression peripherally, as measured by gene expression in circulating monocytes [164]. Specifically, *PER1*, *PER2*, *PER3* and *TEF* gene expression in blood monocytes before and after switching from high carbohydrate/low fat food to low carbohydrate/high fat isocaloric food all showed significant time:diet interactions. Interestingly, significant correlations were established between *PER1* expression changes and total LDL cholesterol in the plasma, *PER2* and plasma non-esterified fatty acid (NEFA) levels, and *TEF* and nuclear factor of kappa light polypeptide gene enhancer in B cells inhibitor, alpha (*NFKB1a*) and *ACOX3* (acyl-Coenzyme A oxidase 3, pristanoyl) changes [164].

Glucose metabolism is highly circadian [165] and depends heavily on the timing and constitution of nutrient ingestion. The extent to which the clock is affected by carbohydrates largely depends on the carbohydrate composition. Highly digestible carbohydrates appear to have stronger entraining capacity than those that are poorly digestible and can induce rapid circadian entrainment in a tissue-specific way [166], [167]. Simple sugars increase levels of fatty acids and glucose in blood, producing a concomitant rise in NAD(P)H levels, which directly influences the efficiency of CLOCK:BMAL1DNA binding. Several important proteins in the regulation of the glucose homeostasis including PEPCK and pyruvate dehydrogenase kinase, isoenzyme 4 (PDK4) are regulated at different levels by the clock genes CRY1 and CRY2 and the metabolic genes PPARα and PPARγ, providing additional linkage between the molecular clock and regulation of glucose metabolism [168], [169].

The effect of sugar and other nutrient metabolism on the circadian oscillator (and vice versa) has been well studied in plants (reviewed in Ref. [170]), but not all studies have been recapitulated in mammalian systems. However, in mammals, fat soluble vitamins (A,D,E,K) exhibit daily rhythmicity in serum with a diurnal peak between 15:16 and 18:08 h [171] and some water soluble vitamins (B and C) are also regulated in a circadian manner and display oscillations in certain tissues examined to date and under certain dietary regimes [9], [26], [121] (also see: circadiomics.igbuci.edu).

A specific set of molecules receiving attention from a circadian perspective during the last decade is polyphenols, which are found frequently in certain foods and possess a wide range of beneficial metabolic effects. Several polyphenols have been shown to have a direct effect on the core clock. Specifically, the unique polyphenols found capable of modulating the molecular clock to date are resveratrol and the proanthocyanidins. The main source of resveratrol in the human diet is red wine (but it is also present in smaller amounts in blueberries, peanut butter, dark chocolate, among other sources) [172]. Resveratrol is thought to increase SIRT1 activity through a direct allosteric activation via the N-terminal activation domain in SIRT1 [173]. SIRT1 is implicated in the prevention of many age-related diseases such as cancer, Alzheimer's disease, and type 2 diabetes [174], controlling, at the cellular level, DNA repair and apoptosis, inflammatory pathways, insulin secretion, mitochondrial biogenesis and the circadian clocks [175]. When administered to animals (gray mouse lemurs), resveratrol can adjust the circadian rhythms of locomotor activity and body temperature [176]; in rats, it can reverse adipose tissue-specific circadian gene expression changes induced by a high fat diet [177], confirming that the circadian system is a target for this polyphenol.

Procanthocyanidins (PAs) are the most consumed polyphenols in human diet based on their widespread presence in vegetables, fruits, cacao, nuts and some beverages like red wine or tea [178]. PAs have protective properties against metabolic syndrome and cardiovascular diseases [179], [180], [181], [182], [183], although some have also been shown to have anticancer properties as well as neuroprotective effects [184], [185], [186]. Related to the molecular clock machinery, proanthocyanidins modulate the expression level of clock-core and clock-controlled genes in peripheral clocks of healthy and obese rats [187] and the 24-h rhythm expression of clock-core and clock-controlled genes in liver and hypothalamus, which is associated with variations of the circadian fluctuation of some important metabolites in plasma or NAD^+^ levels in liver. However, the zeitgeber time of proanthocyanidin administration controls the precise effect of this polyphenol on the molecular clock *in vivo* [28], [188].

Most of the studies to this point show the response of the clock to diets of largely different macronutrient composition. Yet, unclear is whether the response of the circadian clock to a specific diet is due to particular signaling pathways (i.e. impaired insulin sensitivity in the context of a high fat diet) vs. specific metabolites or micronutrients within the diets affecting the clock components more directly. Based on emerging data addressing this question, both indirect and direct mechanisms likely are responsible for clock manipulation *in vivo*. Studies beginning to look carefully at this distinction clearly provide examples of specific nutrients that profoundly alter the clock machinery directly. For example, recent discoveries include the effect of polyamines on the circadian clock. In this case, rather than directly modifying the CLOCK:BMAL1 heterodimer, polyamines directly affect the efficiency of the PER2:CRY1 interaction [189]. The period shortening effect of polyamines on the clock at the physiological level can be observed by simply supplementing polyamines in the drinking water of animals and such supplementation can also restore normal periodicity in aged animals. Heme is another molecule that directly affects the transcriptional complex of the BMAL1:NPAS2 heterodimer [190]. Heme also directly binds to REVERBα [191], [192] and induces a repression of target gene expression. Thus, while changes in pathways such as the insulin signaling pathway can modulate circadian protein activity [193], some molecules such as those described here can bind to or modulate clock proteins directly.

## 6 Circadian solidarity across tissues: are nutrients important for synchronization?

As substantiated by numerous human and rodent studies, synchronicity between clocks appears to be important for metabolic health. Examples of this are found in studies showing the strong association of night shift or rotating shift working humans with increased adiposity, type-2 diabetes, and even cardiovascular disease (reviewed in Refs. [97], [194]). Paradigms employing night “work” or rest phase eating in rodents support these epidemiological studies [103], [195]. Thus, that the light-entrainable pacemaker of the brain is out of sync with the food-entrainable oscillators of peripheral tissues appears to be disadvantageous for energy balance. But what is synchronicity in the first place and what is known about communication across tissues that regulates tissue-specific clock function?

Studies are starting to address this complex question by asking what circadian signals arise from one tissue that assist in entraining or maintaining circadian rhythms in other tissues. For example, phosphatidylcholine 18:0/18:1 (PCI) has been identified in mouse serum to undergo a pronounced PPAR δ-controlled rhythmicity, which is essential for PPARα-mediated fatty acid uptake by the muscle [196]. A high fat diet interferes with the robust rhythmicity of PCI release, increasing serum triglycerides and decreasing uptake of free fatty acids by myocytes. In addition to coordination of the muscle and liver clock by circulating PCI, metabolites secreted by adipose tissue are also implicated in synchronicity required for metabolic control. Studies using mice with an adipose-specific depletion of *Bmal1* show that hypothalamic sensing of circulating polyunsaturated fatty acids (PUFAs) and non-esterified polyunsaturated fatty acids is disturbed when the adipose clock is impaired, a situation which results in altered circadian energy intake and obesity in mice [197]. Thus, temporal coordination of adipocyte and hypothalamic clocks also appears to be essential in clock coordination and is directly linked to the levels of circulating PUFA in the body. Finally, FGF21, a hepatokine that can cross the blood–brain barrier is another example of a nutrient-driven factor that can influence synchronicity across tissues. Specifically, FGF21, which is upregulated during starvation, has recently been shown to target the SCN, where it can alter circadian locomotion in free running conditions [198]. Feeding of animals with a ketogenic diet can mimic the starvation-induced increase in circulating FGF21 and thereby affect circadian rhythms centrally.

**Figure 4:**
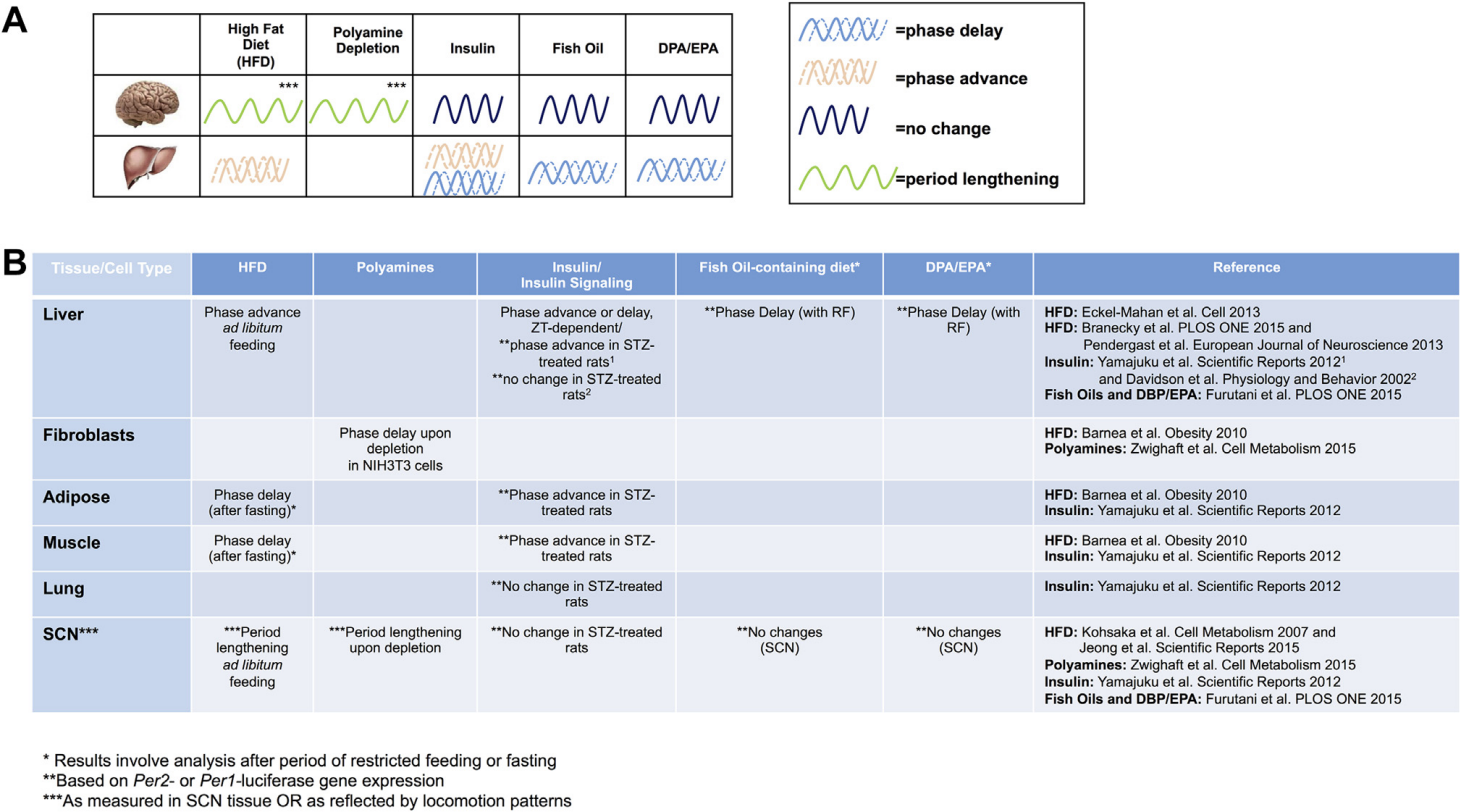
**Overall diet and components of specific diets function as circadian zeitgebers in tissue-specific ways**. (A) HFD (60% kcal from fat), polyamines (often found in vegetables, fruits, cheese, and meat), insulin (to which sensitivity is altered after HFD), and fish oils have been demonstrated as containing zeitgeber properties and can affect the period or phase of existing circadian oscillations in a context-dependent manner. While impaired insulin signaling has been reported to phase-advance the liver clock under some conditions [9], [28], [156], [161], [162], [167], [188], [189], [247], [248], administration of insulin can phase advance or delay the hepatic clock depending on the zeitgeber time of administration. (B) HFD functions as a zeitgeber for multiple tissues, but phase advance or delay results may depend on the post-diet treatment paradigms. (For example, phase delay results in the adipose and muscle tissues may be influenced by the post HFD fasting period prior to tissue analysis [247]). Polyamine depletion has a similar effect on periodicity in NIH3T3 cells and lengthens the period of the central pacemaker (as measured by locomotion analysis) [189]. Impaired insulin signaling by Streptozotocin (STZ) treatment has been shown to phase advance the clock in insulin-sensitive but not less insulin-sensitive tissues [156] but other studies show no STZ-induced phase change without the implementation of restricted feeding paradigms [248]. Diets supplemented with fish oil from various marine organisms or DPA/EPA generally phase delay the liver when administered at ZT0 for several days. Alternatively, the brain clock remains immune to phase or period changes following such a regimen [167].

One important discovery regarding the effect of nutrients on tissue-specific clock response is that in specific circumstances (the results can vary depending on the strain and age of mice, as well as the specific dietary components) a high fat diet can induce obesity simply by disrupting the circadian rhythmicity of energy intake, inducing increased energy intake during the normal resting phase [158], [159], [160]. The molecular basis for this isn't entirely clear, but that the central pacemaker and peripheral clocks such as the liver have an impaired phase relationship is likely based on a phase-advance of the liver after HFD feeding vs. a period lengthening effect of the diet as measured by free-running locomotion. The basis for this interpretation is that a high fat diet has been shown to increase period length at the level of the circadian pacemaker, as measured by wheel running in free running conditions; whereas several studies clearly demonstrate a pronounced phase-advance of the liver clock by a high fat diet (Figure 4) [9], [162]. It is likely that synchrony between peripheral metabolic tissues is also impaired under such conditions; however, studies addressing phase-relationships across tissues are needed and just beginning to emerge. For example, under nutrient stress, serum metabolite oscillations are a poor representative of the clock response in the liver [199], where the clock is reprogrammed in a manner which includes widespread *de novo* rhythmicity in a number of metabolic pathways [9]. Presumably, impaired phase relationship of clocks can occur in humans in response to a high fat diet, but this has not been directly tested. Interestingly, rodent studies suggest that specific high fat diets may have more than transient effects on circadian rhythms as reversal from a high fat diet back to control diet or vivarium chow can produce a rapid return of the liver clock to its prior schedule [9], [162] as well as the normal circadian rhythmicity in eating patterns [162]; however, the return to normal circadian locomotion patterns is variable [9], [162]. The implications of this result for human health remain to be seen, but the results suggest that even transient disruptions of the clock by nutrients may produce more long lasting effects than currently appreciated, at least in some individuals.

## 7 Lipid ligands and nuclear receptors involved in circadian timekeeping

Much of the molecular glue connecting circadian rhythmicity and metabolism comes in the form of nuclear receptors (NR), which connect endocrine and circadian physiology. At the level of gene expression, over half of the 49 mouse nuclear receptors display rhythmic mRNA accumulation [31]. In addition, some of the nuclear receptors that comprise the PPAR, ROR or REV-ERB families, directly participate in one or more of the transcription–translation feedback loops that regulate the clock machinery (Figure 1A). Other nuclear receptors have been shown to potentially interact with some of the components of the clock system directly. For example, the nuclear receptors NURR1 and TRα, among others physically interact with the PER2 protein [200]. HNF6α, a nuclear enriched liver factor, directly interacts with REV-ERB-α [95] and the retinoic acid receptors RAR and RXR interact directly with CLOCK [201].

Natural ligands have been discovered for roughly half of the nuclear receptor superfamily, the majority being lipidic structures [202] often found in the human diet. For example, cholesterol, cholesterol sulfate and oxysterols are natural ligands for RORα and γ, while stearic acid and all-trans-Retinoic acid (ATRA) are ligands for RORβ [203]. Together with ATRA, vitamin A and its derivatives are natural ligands for RARs [204], and 9-cis retinoic, docosahexaenoic acid and phytanic acid are ligands for RXRs [204], [205]. In the case of the PPAR family, fatty acids, eicosanoids and oleylethanolamide, a metabolite found in the gut, have been classified as their natural ligands [206]. Table 3 presents a number of additional ligands identified for various members of the nuclear receptor family with known circadian roles. (In instances where synthetic ligands have been developed for a particular nuclear receptor, these are also listed.) Finally, oxysterols and phytosterols such as stigmasterol, sisterol and campesterol have been shown as natural ligands for LXR [205], [207], [208], a direct nuclear receptor target of SIRT1 [209]. Thus, by responding to specific lipid ligands, nuclear receptors are a key point of integration for nutrient metabolism and the clock, and provide key temporal information for the necessary transcriptional and associated chromatin machinery.

## 8 Potential for diet as a modulator of human metabolic health via clock regulation

While new studies considering the role of the human diet and nutrition on our circadian clocks (and vice versa) are emerging, these data lag far behind the numerous rodent studies demonstrating a direct role for nutrients in circadian biology. Since the introduction of artificial light and nighttime work, serious health consequences have been reported for those who sleep less, work night or rotating shifts, or have social jetlag resulting from work or social schedules that are not in sync with the external light/dark cycle. Association studies have revealed that such circadian misalignment is linked with impaired glucose tolerance, reduced insulin responsiveness following glucose challenge, increased body mass index, decreased levels of leptin, and increased levels of ghrelin [214], [215], [216], [217] and metabolic syndrome [218], [219], [220]. In addition, social jetlag and similar circadian misalignment result in increased BMI [214], [221]. One compelling clinical study designed to examine the role of circadian alignment on metabolic physiology comes from an experimental paradigm in which healthy volunteers were placed on a 28-h “day” and scheduled to sleep at different phases throughout the circadian cycle. When subjects were shifted 12-h from their normal sleep/wake cycle, they exhibited decreased leptin, increased glucose, and elevated blood pressure. In addition, their post-meal glucose response was similar to that seen in pre-diabetic patients [222]. Together, these studies highlight the detrimental health effects of disrupting the circadian system in humans by means of a desynchronization with the light/dark cycle. Considering that metabolic disease is associated with circadian disruption in humans [214], [215], [216], [217], [218], [219], [220] and that the clock uses nutrient input to set the local time, it is conceivable that both the timing and nutrient composition of one's diet might be key components of personalized medicine's future, in parallel to individual behavioral and genetic predisposition.

While additional studies are needed, one way to include behavioral circadian predisposition into personalized medicine may be to ascertain the circadian chronotype (essentially, the sleep/wake preferences and patterns of a subject) of patients, which may predict the effectiveness of a given treatment. Humans display great circadian variability at the behavioral level with some individuals having more ‘eveningness’ (a delayed sleep onset) compared to those with more ‘morningness’. To determine chronotype, questionnaires are often used, the most common one being the ‘Morningness–eveningness questionnaire’ of Horne-Östberg (originally based on rhythmicity in body temperature) [223] and the Munich chronotype self-rated scale questionnaire [224]. In general, the results of both questionnaires present the level to which an individual's internal clock adjusts to their environment using a combination of sleep logs and actimetry. The reliability of such tools has been demonstrated in a number of studies. One such study assigned chronotype to 95 individuals, using the Munich chronotype questionnaire. In this study, people identified as having increased ‘morningness’ vs. ‘eveningness’ were assayed for saliva and oral mucosa every four hours to study circadian melatonin levels and clock gene expression, respectively. Importantly, a significant correlation between the phase of melatonin secretion and the sleep time confirmed the patient's reported chronotype. Similarly, the expression phase of several clock genes was found advanced or delayed in a manner which correlated with individual chronotype [225]. Taking into account how timing and nutrient composition of diet can modulate the clock, the identification of chronotype may in the future be an important determination in the clinic. For instance, having dinner or snacking late, sleeping fewer hours and eliminating breakfast are usual trends for the ‘eveningness’ chronotype, and may be useful information when performing lipid profiling, etc. [153], [154], [226], [227], [228], [229]. Understanding when behavioral vs. pharmacological intervention is possible based on chronotype might expand treatment options for specific metabolic disorders.

A defined chronotype in humans is ultimately based on the same circadian clock mechanisms identified in cells and tissues of other mammals. For example, the circadian expression pattern of a majority of clock genes has been observed in RNA samples from human peripheral tissues including the skin, oral mucosa, colonocytes, white blood cells, peripheral blood mononuclear cells, pineal gland tissue and visceral and subcutaneous white adipose tissue [230], [231]. As clock components are conserved in humans, candidate mutations in clock genes that may affect their function are being searched for in humans. Recently, a number of single nucleotide polymorphisms (SNP) in some of the clock genes have been associated with impaired metabolic and physiological phenotypes in humans. For example, associations between particular mutations in the clock components CKδ, PER3 and PER2 with circadian sleep disorders have been made [232], [233], [234], [235], and SNPs in the core clock genes *PER2*, *CRY1*, *REV-ERBα* and *CLOCK* have been associated with metabolic disturbances. Specifically, *PER2* SNPs rs2304672C>G and rs4663302C>T have been associated with abdominal obesity. Interestingly, rs2304672 C>G minor allele carriers have a greater probability of displaying extreme snacking, experiencing stress with dieting, eating when bored, skipping breakfast and dropping out of a dietary treatment of obesity than non-carriers, while frequency of the rs4663307 minor allele is increased in withdrawers than in those subjects who successfully completed treatment [236]. Homozygous individuals with the minor C allele of the *CRY1* rs2287161 polymorphism have been associated with an increase in carbohydrate intake (%of energy intake), HOMA-IR and fasting insulin [237]. The T minor allele of the REV-ERB-α rs2071427 polymorphism is associated with a higher BMI [238] while minor allele carriers for the REV-ERB-α rs2314339 polymorphism have a lower probability of abdominal obesity than non-carriers, as they have lower obesity parameters and lower waist circumference values. From this group, carriers of the minor allele (AA + AG) have greater protection against obesity than GG homozygotes, when they also have a high intake of MUFA (≥55% of total fat) [239].

Out of the identified SNPs in clock genes, the *CLOCK* gene SNPs have been the most comprehensively studies. Several of these SNPs are associated with obesity and individual components of the metabolic syndrome. SNP rs4580704 is associated with reduced BMI and compared to non-carriers, minor allele carriers have a 31% lower risk of diabetes, 45% lower risk of having hypertension, lower postprandial triglyceridemia after a fat-loading test, lower intake of fat, and lower plasma glucose concentrations and HOMA values when MUFA intake is the same or more than 13.2% of energy [240]. SNP rs3749474 is associated with a higher BMI and compared to non-carriers, minor allele carriers show a higher intake of fat in the diet. In addition, minor allele carriers carrying at least one copy of the T allele, display a higher degree of obesity (weight and BMI) and abdominal obesity (waist circumference) than major allele carriers [241]. Compared to non-carriers, SNP rs1801260 (3111T/C) minor allele carriers have larger waist circumferences when the saturated fatty acid intake is bigger or equal to 11.8% of energy. In addition, those subjects are less successful losing weight under a weight reduction program of 28 weeks [241]. After 12 months of a low-fat intervention, subjects who are homozygous for the major allele (TT) display lower plasma insulin concentrations, lower insulin resistance and higher insulin sensitivity compared with carriers of the minor allele C (TC + CC) [242]. Finally, the haplotype of rs1554483G and rs4864548A is associated with a 1.8-fold risk of being overweight or obese [243]. In summary, mutations of the human clock genes generally are associated with impaired metabolism. Further studies will likely expand on the nature of and mechanisms underlying this predisposition. Nevertheless, consistent with several large epidemiological studies addressing behaviorally-induced circadian misalignment in humans, normal function of the circadian clock components may be important for metabolic health and balance.

Understanding one's genetic or epigenetic predisposition for circadian disruption may be one of the key aspects of personalized medicine in the future. Several studies have begun laying the groundwork for this. Specifically, a better understanding of the internal clock in humans at the level of circulating metabolites is gaining ground, with efforts to identify internal body time [244]. Multiple studies have been performed to show that the metabolome oscillates throughout the circadian cycle [30], some of which are completely independent of the eating cycle. In a study that involved 40 h of constitutive wakefulness and isocaloric hourly feeding in constant illumination conditions, numerous metabolites (approximately 15% of those measured) were found to still oscillate in both plasma and blood samples when measured by hourly collection. In spite of no rhythmicity in feeding, lipid species were generally higher during the light phase (mid-morning to noon) while some other metabolite pools (such as amino acids) were more equally distributed in their circadian peak around the 24-h cycle [30]. Thus, without sleep or daily variation in food intake, approximately 15% of metabolites cycle in humans. A much greater percentage of metabolites oscillate when rhythmicity in sleeping and eating occurs [29], an observation that has been repeated in rodent studies [26]. In fact, in rhythmic eating and sleeping conditions, between 60 and 70% of all metabolites observed in human plasma oscillate throughout the circadian cycle [29]. In Davies et al., meals were administered 3× day (with one snack) and diet macronutrients were reflective of UK dietary guidelines, with an ultimate consumption of 35% fat, 49% carbohydrate, and 12% protein. When diet and sleep were maintained and aligned with the light/dark cycle, the majority of plasma metabolites remained oscillatory. Across four studies, which varied substantially in the extent to which sleep and food consumption were controlled, a number of lipid species has been shown to oscillate throughout the circadian cycle. This number increases dramatically when not comparing across individuals, demonstrating that individual variation in the phase of oscillating lipids does exist. Nevertheless, these data underscore the importance of diet-derived nutrient signals that circulate, are known to influence the clock at the level of nuclear receptors and proteins, and that can orchestrate circadian control in a tissue-specific way.

## 9 Conclusions

While the model of personalized medicine, the idea of customizing healthcare based on parameters such as the genetic and behavioral profiles of an individual, once seemed a thing of the future as it related to nutrient input, some key studies already show its tremendous promise. For example, machine-learning algorithms can use blood parameters, nutrient input choices and gut microbiota composition to help predict glycemic responses to a meal [245]. While one meal might produce a particular rise in postprandial blood glucose in one individual, the same meal might produce an entirely disparate glycemic response in another. It is anticipated that like gut microbiota or genetic SNPs, one's internal clock phase will be as integrated into personalized medicine as the rest. For example, when coming to the clinic, routine questions might consider sleep duration, chronotype, and the extent of one's social jetlag. Even social jetlag (independent of shift work) has proved to be associated with increased BMI, fat mass, waist circumference, obesity, and metabolic syndrome, but varying in healthy obese vs. unhealthy obese individuals [214]. As the number and identity of clock gene SNPs are further investigated, this may be another mechanism by which nutritional and/or pharmacological regimens are recommended for a patient.

Already, practical applications for the general public are being developed based on decades of research on the internal clock and its molecular underpinnings. For example, the Entrain app was developed based on the heavy dependence of our internal clock on the light/dark cycle. This app was designed to assist with optimal circadian reentrainment to a new light/dark cycle using mathematical models based on and incorporating thousands of different circadian misalignment possibilities [246]. Nutrition has also been proposed as a powerful reentrainment mechanism to circadian misalignment as well, based on the repeated demonstrations of its effectiveness in controlling peripheral circadian rhythmicity [6], [7], [8]. Based on the number of potential zeitgebers that arise from different nutritional inputs, it may be that food intake and chronotype will jointly play a prominent role in the future of personalized medicine.

In summary, what is clear is that circadian rhythms are important for energy balance at the cellular and organismic level. Importantly, feeding is a critical modulator of the internal clock and, in addition to affecting synchronicity between the central pacemaker and metabolically active peripheral tissues, it likely controls the extent to which peripheral tissues are in phase with one another. With this in mind, several questions remain: 1) how does synchronization across tissues take place, 2) what are the specific nutrients that modulate tissue-specific rhythms in a way that preserve synchronization across the body, and 3) can we use nutritional input more effectively in dealing with metabolic disorders arising from circadian misalignment? The complexity of the interplay between nutrient metabolism and circadian rhythmicity remains, but understanding this dialog more fully is likely to eventually produce great benefits in terms of metabolic disease prevention and treatment as it relates to circadian misalignment.

## Figures and Tables

**Table 1:** Examples of metabolic CLOCK-BMAL1 target genes.

Do not directly affect the function of one of the TTFL circadian loops	Directly affect the function of one of the TTFL circadian loops
*Alas1*, *Pai-1*, *Trα*, *Tef*, *Hlf*, *Hmgcr*, *Abcc2*, *Anpep*, *Abcb1a*, *Scl22a23*, *Scl22a2*, *Prkab1*, *Slco2b1*, *Scl22a5*, *Scl16a10*, *Agtr1a*, *Sclco1b2*, *Car12*, *Cyp2b10*, *Oprt*, *Scl22a6*, *Ntrk2*, *Esr1*, *Egfr*, *Hsp90aa1*, *Hsp90b1*, *Mtrr*, *Tubg1*, *Htr2a*, *Adra1b*, *Pah*, *Cbs*, *Pdxk*, *Adra1b*.	*Pparα*, *Nampt*, *Dec1*, *Dec2*, *Errα*, *Prox1*, *Dbp*.

**Table 2:** Examples of tissue functions under circadian control.

Tissue	Main pathways (examples of central genes under circadian control)	References
Liver	Gluconeogenesis (*Pepck*, *Sirt1*, *Pgc-1α*, *Foxo1*, *Torc2*, *Shp*) Glycolysis (*Pgc-1α*, *Sirt1*, *Pfkm*, *Pklr*) Glycogen synthesis (*Gck*, *Gsk3β*) Glycogenolysis (*Pygl*, *G6pc*) Fatty acid β-oxidation (*Cpt1*, *Pgc-1α*) Cholesterol synthesis (*HmgCoAR*, *Fxr*, *Lxrα*, *Srebp-1c*) Lipogenesis (*Acac*, *Fasn*) Ketogenesis (*Foxa 2*, *Hmg-CoA*) Synthesis of urea (*Otc*, *Arg1*, *Cps1*, *Ass1*, *Asl*) Synthesis of bile acids (*Cyp27a1*, *Fgfr4*, *etc* (*See references*.)) Xenobiotic metabolism (*Car*)	[123], [124], [125], [126], [127], [128], [129], [130], [131], [132], [133], [134], [135]
Heart	β-oxidation (*Mcad*, *Lcad*, *Hadhα*) Krebs cycle (*Cs*, *Idh*, *Ogdh*) Blood circulation (*Agt*) Angiogenesis (*Vegfa*, *Flt1*, *Kdr*)	[14], [136], [137]
Skeletal muscle	Glycolysis (*Pgc-1α* – *Sirt1*, *Hk*, *Pfkm*, *Pkm*) Glycogen synthesis (*Hk*, *Gsk3β*) Glycogenolysis (*Pygm*, *G6pc*) Fatty acid β-oxidation (*Cpt1*, *Pgc-1α*)	[126], [138], [139]
Kidneys	Renal sodium balance and electrolyte reabsorption (*Usp2*, *Gilz*, *Slc2a9*)	[140]
White adipose tissue	Fatty acid esterification (*Fas*) Lipolysis (*Hsl*) Lipogenesis and adipogenesis (*Acac*, *Fasn*, *Pparγ*, *Rev-erbα*, *C/ebp*)	[141]

**Table 3:** Natural and synthetic ligands of nuclear receptors with circadian function.

Nuclear receptors	Natural ligands	Synthetic ligands	References
LXR	**Oxysterols:** 24(S)-hydroxycholesterol (brain) 22(R)-hydroxycholesterol (adrenal) 24(S),25-epoxycholesterol (liver) 27-hydroxycholesterol (human macrophage) **Phytosterols:** Stigmasterolsitosterolcampesterol	T0901317 GW3965	[205], [207], [208]
LXRα	**Intermediary cholesterol biosynthesis:** 4,4-dimethyl-5a-cholesta-8,14,24-trien-3b-ol		[208]
RXR	9-cis retinoic Docosahexaenoic acid Phytanic acid	Bexarotene PNCR	[204], [205], [210]
RAR	Vitamin A and derivates All-trans-Retinoic acid (ATRA)	Arotinoid acid Am 580 BMS209641 CD666 BMS270394 BMS204493 AGN193109 BMS195614 Ro-41-5253 PNCR	[204], [210]
PPARα	Docosahexaenoic acid Eicosapentaenoic acid Leukotriene B4 8-hydroxy-eicosatetraenoic acid	Fenofibrate Clofibrate Gemfibrozil	[211]
PPARγ	Docosahexaenoic acid Eicosapentaenoic acid MUFA Arachidonic acid metabolites Triterpenoids	thiazolidinediones	
PPARβ/δ	Long-chain fatty acids Carboprostacyclin Components of VLDL	GW501516	
RORα	Cholesterol Cholesterolsulphate 7α-hydroxycholesterol 7β-hydroxycholesterol 7-ketocholesterol 20α-hydroxycholesterol 22*R*-hydroxycholesterol 25-hydroxycholesterol 24*S*-hydroxycholesterol 24*R*-hydroxycholesterol 24,25-epoxycholesterol Neoruscogenin (25*S*)-ruscogenin	T0901317 SR1078 SR3335 SR1001	[203]
RORγ	7α-hydroxycholesterol 7β-hydroxycholesterol 7-ketocholesterol 24*S*-hydroxycholesterol 24*R*-hydroxycholesterol 24,25-epoxycholesterol	T0901317 SR1078 SR2211 SR1555 SR1001 Digoxin Ursolic acid ML209 *N*-(4,6-dimethyl-benzo[d]thiazol-2-yl)-3-methyl-thiophene-2-carboxamide *N*-(2-(4-ethyl-phenyl)-2*H*-benzo-[d][1,2,3]triazol-5-yl) propionamide *N*-(5-benzoyl-4-p henylthiazol-2-yl)-2-(4-(ethylsulfonyl) phenyl)acetamide	
RORβ	Stearic acid ATRA		
NURR1	In NURSA there are not ligands	6-mercaptopurine Isoxazolopyridinone	[212], [213]
TRα	Thyroid hormone	PNRC GC-1	[210] NURSA
ERRα		XCT790 Octochorocamphene	NURSA
SIRT6 (and other sirtuins)	Myristic, oleic and linoleic acids		[145]
